# Global gene expression analysis reveals reduced abundance of putative microRNA targets in human prostate tumours

**DOI:** 10.1186/1471-2164-10-93

**Published:** 2009-02-26

**Authors:** Ruping Sun, Xuping Fu, Yao Li, Yi Xie, Yumin Mao

**Affiliations:** 1 State Key Laboratory of Genetic Engineering, Institute of Genetics, School of Life Sciences, Fudan University, Shanghai, PR China

## Abstract

**Background:**

Recently, microRNAs (miRNAs) have taken centre stage in the field of human molecular oncology. Several studies have shown that miRNA profiling analyses offer new possibilities in cancer classification, diagnosis and prognosis. However, the function of miRNAs that are dysregulated in tumours remains largely a mystery. Global analysis of miRNA-target gene expression has helped illuminate the role of miRNAs in developmental gene expression programs, but such an approach has not been reported in cancer transcriptomics.

**Results:**

In this study, we globally analysed the expression patterns of miRNA target genes in prostate cancer by using several public microarray datasets. Intriguingly, we found that, in contrast to global mRNA transcript levels, putative miRNA targets showed a reduced abundance in prostate tumours relative to benign prostate tissue. Additionally, the down-regulation of these miRNA targets positively correlated with the number of types of miRNA target-sites in the 3' untranslated regions of these targets. Further investigation revealed that the globally low expression was mainly driven by the targets of 36 specific miRNAs that were reported to be up-regulated in prostate cancer by a miRNA expression profiling study. We also found that the transcript levels of miRNA targets were lower in androgen-independent prostate cancer than in androgen-dependent prostate cancer. Moreover, when the global analysis was extended to four other cancers, significant differences in transcript levels between miRNA targets and total mRNA backgrounds were found.

**Conclusion:**

Global gene expression analysis, along with further investigation, suggests that miRNA targets have a significantly reduced transcript abundance in prostate cancer, when compared with the combined pool of all mRNAs. The abnormal expression pattern of miRNA targets in human cancer could be a common feature of the human cancer transcriptome. Our study may help to shed new light on the functional roles of miRNAs in cancer transcriptomics.

## Background

MicroRNAs are endogenous, approximately 22 nt single-stranded non-coding RNAs that negatively regulate protein expression by binding to the 3' untranslated regions (3' UTR) of messenger RNAs (mRNAs) and inhibiting translation or inducing mRNA degradation or deadenylation [1]. MiRNA genes are expressed as large precursor RNAs, called pri-mRNAs, which may encode multiple miRNAs in a polycistronic arrangement. These precursors are converted into mature miRNAs of 19 to 25 nucleotides by the RNase III enzymes, Drosha (nuclear) and Dicer (cytosolic). MiRNAs have been identified in the genomes of plants, animals and viruses. Human miRNAs have been implicated in a variety of biological processes, and it is estimated that 30% of protein coding genes are regulated by miRNAs [2].

Recently, the potential role of miRNAs in human cancers has been indicated by several studies, which suggest that aberrations in miRNA expression in cancers may be involved in tumour genesis and progression [3]. Abnormal expression of miRNAs has been found in various cancers, and profiling of miRNAs has been shown to be a more accurate method of classifying cancer subtypes than profiling protein-coding genes. In the case of prostate cancer, a highly prevalent disease in the western world, several miRNA expression profiles have been reported [4-8]. Although some of these studies are not consistent, potentially due to differences in samples or chip platforms, all of them confirmed the widespread dysregulation of miRNAs in prostate cancer. However, the disruption of miRNA expression observed in human cancers needs to be understood by analysing the causes and consequences of the miRNA alterations. Thus far, the causes of miRNA dysregulation are partially known to be the results of three mechanisms: (1) miRNA genes tend to locate in cancer related genomic regions; (2) miRNA expression is epigenetically regulated in cancers; and (3) miRNA processing genes such as *Drosha *and *Dicer *are de-regulated in cancers. However, little is known about the consequences of improper regulation of miRNA expression. More work needs to be done to show whether these miRNAs have a direct function in cancer progression or are simply differentially modulated in tumours.

Identifying the genes targeted by miRNA is crucial for understanding the functions of the miRNAs. Based on the conservation of 3' UTRs, which are complementary to the "seed" region (nucleotides 2–7 from the 5' end) of miRNAs, several computational methods have been developed to search for miRNA targets. Some of these methods have been biologically validated and proven to be accurate [2,9]. According to single miRNA-mRNA target pair information, miRNAs are thought to function as either tumour suppressors or oncogenes. For example, the *let-7 *family has been shown to target the *RAS *gene [10]. Down-regulation of the *let-7 *has been found in lung cancer, and this finding is correlated with a poor prognosis. Thus, reduced expression of *let-7 *is predicted to promote lung cancer progression. However, the situation is complicated by the fact that miRNAs regulate multiple genes, and a single mRNA can be targeted by several different miRNAs. Therefore, the impact of changes in miRNA expression in cancers is likely to be dependent on the cellular context.

Evidence is emerging that miRNAs can not only repress translation of mRNAs but can also induce their degradation, even if the mRNA target sites have only partial complementarity to the miRNAs. For example, several studies have revealed a correlation between the expression of miRNAs and that of their targets through analysis of mRNA target gene expression profiles and in situ hybridisation [11-13]. A recent publication has reported the impact of miRNAs on global mRNA and protein expression and showed that the regulation of protein-coding genes by miRNAs is quite similar at both the transcript and protein levels [14]. Moreover, Wu et al. have reported that miRNAs increase target mRNA decay rates by promoting rapid deadenylation [1]. Assuming that the miRNAs dysregulated in prostate cancer have an influence on the expression of their targets, analysis of target gene expression may provide clues to the functions of miRNAs in prostate cancer.

In this study, we computationally explored the global expression patterns of miRNA targets in human prostate cancer using several published microarray datasets. Interestingly, in contrast to all the mRNAs with altered expression in prostate cancer relative to benign prostate tissue, the transcript levels of miRNA targets were significantly lower. Closer examination revealed a positive correlation between the reduced abundance of miRNA targets and the number of target-site-types in the 3' UTRs of the target mRNAs. Remarkably, we found that the predicted targets of the up-regulated miRNAs in prostate cancer, reported by a miRNA profiling study, were significantly more likely to be down-regulated in prostate tumours than the predicted targets of all other miRNAs. We also found that the transcript levels of miRNA targets were lower in androgen-independent prostate cancer than in androgen-dependent prostate cancer. Furthermore, the abnormal expression pattern of miRNA targets could be a common feature of the human cancer transcriptome.

## Results

### Globally reduced transcript levels of miRNA targets relative to total mRNAs in prostate tumours

**Figure 1:**
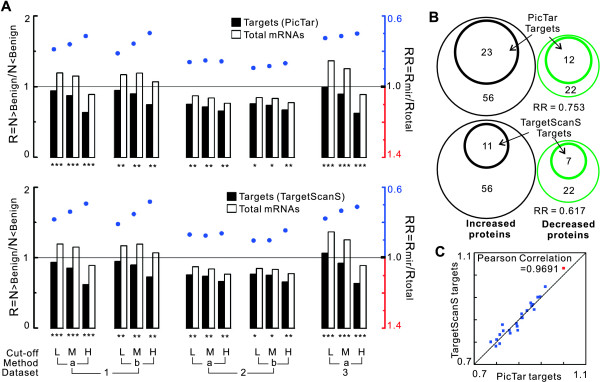
**Comparison of transcript levels of miRNA targets between in prostate cancer and in benign tissues**. (A) The transcript levels of miRNA targets in prostate tumours were compared with those of total mRNAs using R_mir_, R_total _and RR values (see methods). The R values of all miRNA targets (R_mir_) and total altered mRNAs (R_total_) are represented as black and white bars, respectively. Also plotted are the RR (R_mir_/R_total_) values (colored dots, right axis). The RR value is a surrogate for an increased or decreased abundance of miRNA targets relative to total mRNAs. Blue color denotes RR < 1 and red denotes RR > 1. Three gene expression microarray datasets (Dataset 1, 2 and 3) were used for this comparison. Three different cut-off values (L: Low; M: Medium; and H: High) and two methods a and b (for dataset 1 and 2) were chosen to rule out the bias of single method. Asterisk represents the statistical significance of each comparison (resampling statistical test, see methods). One asterisk means *P *< 0.05; two asterisks, *P *< 0.01; three asterisks, *P *< 0.0002. The complete data are reported [see Additional file 1]. (B) Analysis of the protein levels of miRNA targets using a small proteomic dataset. Black and green cycles represent the numbers of increased and decreased proteins in prostate tumours relative to benign prostate tissues, respectively. Small cycles represent the number of miRNA targets mapped into these two protein groups. Areas of the cycles are scaled to the proteins number. (C) Correlation between the RR values obtained using two different miRNA target predictions.

Using the gene expression atlas published by three independent groups (Table 1) [15-17], and the conserved miRNA target predictions from PicTar [9] and TargetScanS [2], we generated a global view of the transcript levels of miRNA targets in prostate cancer. First, the expression values of each mRNA were compared between localised prostate cancer and benign prostate tissue in each dataset, to determine if the mRNA had a higher or lower expression level in the cancer tissue. After sorting total mRNAs into three groups (high expression, low expression and unchanged), we calculated the R_mir_, R_total _and RR values (see Methods). The RR value is a surrogate for an increased or decreased abundance of miRNA targets relative to total mRNAs. Notably, for all three datasets, RR values were less than 1 when comparing localised prostate cancer with benign prostate tissue (Figure 1A and [see Additional file 1]). Resampling statistical tests indicated that the differences were significant (*P *< 0.05). To confirm this observation, we used the Hyper-Geometric distribution to evaluate the enrichment levels of miRNA targets in the three mRNA pools and found a unique and significant enrichment in the low expression pool across all three datasets (Enrichment *P *< 0.05) [see Additional file 1]. Furthermore, the average expression ratio of miRNA targets (0.997) was significantly lower than that of the non-miRNA-target genes (1.021, *P *< 10^-100^, t test, dataset 1). These findings reveal that miRNA targets have a high propensity to fall in the low expression mRNA group in localised prostate cancer, an observation that is robust across different datasets. Similar results were observed when comparing metastatic prostate tumours or all prostate tumours with benign tissues [see Additional file 1]. Through the rest of this study, we focused on the comparison between localised prostate tumour and benign prostate. To determine whether the low expression of miRNA targets occurred in benign tissues, we randomly divided the benign samples from dataset 1 into two classes and undertook a benign-benign comparison. In this case, there was no significant difference between miRNA targets and total mRNAs (*P *> 0.05). The absolute R_mir _and R_total _value variation in the three datasets might result from differences in the chip platforms or from intrinsic differences in the samples. Nonetheless, the fact that RR ratios were always less than 1 for tumour when compared with benign prostate indicates that, in contrast to total mRNA, the transcript abundances of miRNA targets are significantly lower in prostate cancer than in benign prostate tissue.

**Table 1:** Gene expression datasets used in this global analysis

					**Number of samples**		
					
**Cancer** **Type**	**Dataset**	**Refs.**	**Array type**	**Probe** **sets**	**Benign**	**Localised**	**Metastatic**
*Prostate cancer*	1	[15]	Affy^†^	37690	58	66	25
	
	2	[16]	cDNA	26260	41	61	9
	
	3	[17]	cDNA	9984	19	14	20
	
	Correlation analysis	[19]	Affy^†^	22277	10	10	
	
	AD and AI analysis	[21]	cDNA	13314	3	17 AD/8 AI	

*Breast cancer*	4	[33]	Affy^†^	47000	7	40	

*Lung adeno*-*carcinoma*	5	[34]	Affy^†^	7129	10	86	

*Acute myeloid leukaemia*	6	[35]	cDNA	21370	6^‡^	23^§^	

*Liver* *cancer*	7	[36]	cDNA	23075	76	104	

^†^: Affymetrix oligo nucleotide microarray. ^‡^: Normal Bone Marrow. ^§^: Acute Myeloid Leukaemia. Correlation analysis: The dataset used in the correlation analysis between the transcript levels of individual miRNAs and those of their putative targets. AD and AI analysis: The dataset used in the analysis of the transcript levels of miRNA targets in androgen-dependent and androgen-independent prostate cancer.

Given the considerable noise of the gene expression data and miRNA target prediction, we ensured the validity of the above observations using three approaches. Firstly, because microarray results may vary depending on quality of the samples, we collected the gene expression profiles that contained a relatively large number of samples and classified the tissues adjacent to the tumours as benign prostate tissues for all three datasets. Secondly, to rule out the influence of different methods on determining the high, low and unchanged expression groups, two different methods, a and b, were adopted for datasets 1 and 2, where the measurements of matched samples were provided. We also adopted three different cut-off values to determine the mRNAs with altered expression. As shown in Figure 1A, the global low expression of miRNA targets did not vanish when we changed methods or cut-offs, and the RR values actually decreased with the raised cut-offs. For example, in dataset 1, RR = 0.79, 0.76, and 0.71 for low, medium and high cut-offs, respectively (Method a, PicTar targets). Finally, to determine if this observation is robust for different miRNA target predictions, we carried out the same analysis using two different target predictions, which are the leading programs in this field, and obtained similar results. Figure 1C shows that the RR values obtained using the two predictions were highly correlated (Pearson correlation = 0.9691). Additionally, since repression by miRNAs also result in decreased translation, we analysed the protein levels of several miRNA targets using a small proteomic dataset. In this dataset, 64 proteins were reported to be dysregulated in prostate cancer, relative to benign prostate, by employing a high-throughput immunoblot approach [18]. After converting the names of these proteins into RefSeq mRNA IDs (some proteins have more than one RefSeq ID), we mapped all miRNA targets to them. As shown in Figure 1B, the miRNA targets showed a propensity to fall in the decreased protein group (RR < 1), suggesting a reduced protein level of miRNA targets in prostate tumour. Taken together, our data indicate that miRNA target genes are expressed at lower levels in prostate cancer than normal prostate tissue.

### Association between transcript abundance of miRNAs and their target mRNAs in prostate cancer

**Figure 2:**
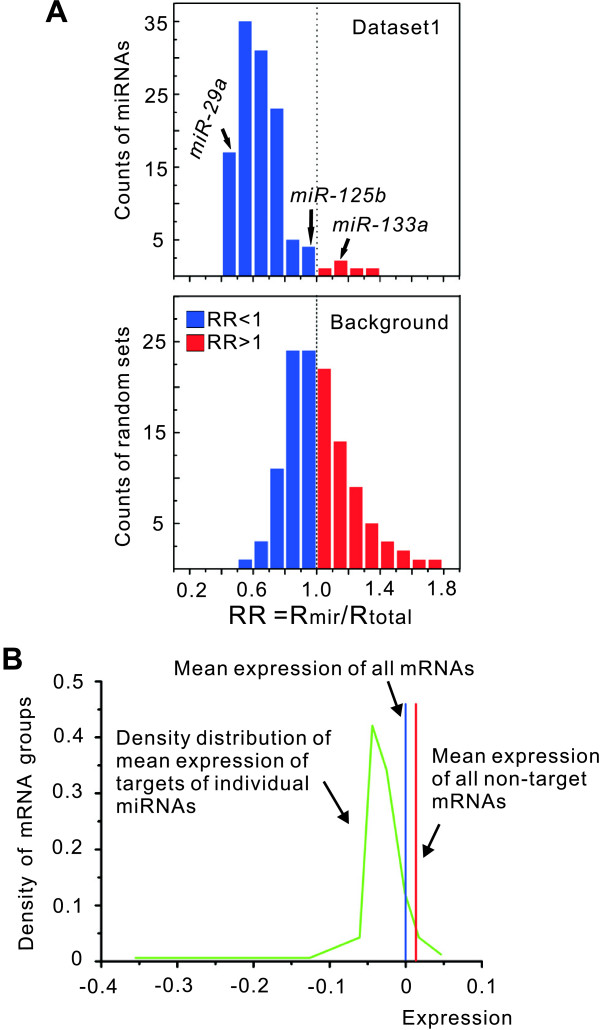
**Transcript levels of the target groups of individual miRNAs in prostate cancer**. (A) Count distribution of RR values of the targets of 120 individual miRNAs is shown above. Count distribution of RR values of random mRNA sets is shown below. Blue color denotes RR < 1 and red denote RR > 1. (B) Density distribution (green line) of mean expression values (log ratio) of the targets of individual miRNAs clearly departed from the mean expression value of total mRNAs (blue line) and that of non target mRNAs (red line).

To further investigate the globally low expression of all miRNA targets, we calculated the RR values of the targets of 120 individual miRNAs. These miRNAs had more than 50 PicTar targets with altered expression (with an average of 125 targets per miRNA) in comparing localised prostate tumours with benign tissues (Method a, Medium cut-off, Dataset 1). If we were to randomly select a set of mRNAs same as the number of targets of each miRNA found in dataset 1, the distribution of RR values generated by the random sets would approximate a background distribution. The distribution of individual miRNAs clearly differed from the background distribution: the RR values obtained for individual miRNAs clustered in the range of 0.4 to 0.8 (Figure 2A), suggesting that these miRNAs may down-regulate their predicted targets in prostate tumours. We also found that the distribution of mean expression values of the target genes of individual miRNAs clearly differed from the mean expression value of total mRNAs (Figure 2B). Among the 120 individual miRNAs studied, *miR-194*, *miR-193 *and *miR-29a *showed the lowest RR values (0.414, 0.423 and 0.435). It should be mentioned that a few target groups preferentially show a relatively high expression level. For example, *miR-133a*, which was recently reported to be down-regulated in prostate tumours [19], had an RR value > 1 (1.194). Similarly, *miR-125b*, which was validated to be down-regulated in prostate cancer by quantitative RT-PCR assays [8], had a relatively high RR value (0.958).

**Figure 3:**
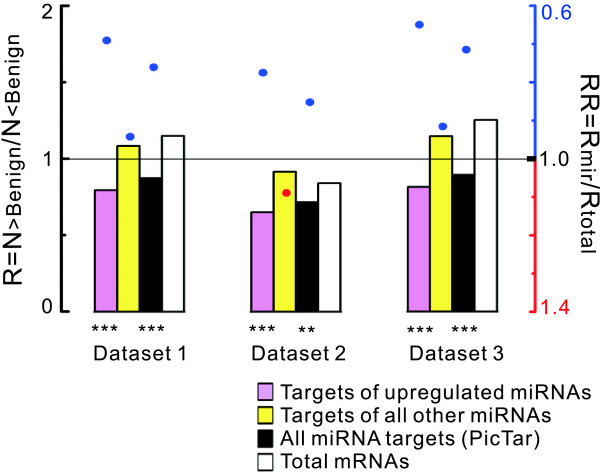
**Transcript levels of the targets of up-regulated miRNAs and of all other miRNAs**. All PicTar targets were divided into two groups. Group I contained the targets of 39 significantly up-regulated miRNAs in prostate cancer reported by Volinia et al. (36 of the 39 had conserved PicTar targets). Group II contained all other miRNA targets. Pink and yellow bars represent R_mir _values of group I targets and group II targets, respectively. Black and white bars represent R values of all miRNA targets and total mRNAs, respectively. RR values and *P *values are analyzed as in Figure 1A.

We next asked whether there was an association between the low expression of general targets and the dysregulation of miRNAs in prostate cancer. Currently, the literature shows that the expression of miRNAs and their targets are expected to be inversely correlated [12]. Namely, the low expression of miRNA targets might imply a concurrently high expression of these miRNAs in prostate cancer. This trend was seen in a miRNA expression profiling study, which showed a significant up-regulation of many miRNAs in prostate cancer [4]. As expected, when relating the expression of miRNA targets to that of the miRNAs using this miRNA expression profile, we found a weak negative correlation between the differential expression scores of individual miRNAs and the RR values of their targets (Spearman correlation, R_s _= -0.342, *P *= 0.025, N = 43) [see Additional file 2]. In contrast to the studies using over-expression (or knockdown) of a single miRNA to detect correlation between the expression of the miRNA and its targets, cancer cells show alterations of many miRNAs with overlapping targets, and thus, it is hard to judge the contributions of individual miRNAs to the low expression of their targets. To facilitate a more global view, we divided all PicTar targets into two groups. Group I contained 5514 targets of 39 significantly up-regulated miRNAs in prostate cancer reported by Volinia et al. (36 of the 39 had conserved PicTar targets). Group II contained all other predicted miRNA targets (2663 targets). After mapping these two groups to the mRNAs with altered expression (Medium cut-off, Method a), we found that the predicted targets of the 36 up-regulated miRNAs were significantly more likely to be down-regulated in prostate tumours than the predicted targets of all other miRNAs (Figure 3, *P *= 0.001, 0.007, 0.013 for Dataset 1–3, respectively). In all three datasets, there was no difference in the relative transcript abundance between mRNA from group II and total mRNAs (*P *> 0.05). The RR values for the putative targets of the 36 up-regulated miRNAs (group I targets) were significantly lower than the RR values for the group II targets in these datasets, indicating a selective down-regulation of group I targets in prostate tumours for all three datasets. We then constructed a gene set containing 8177 genes (the same number of PicTar targets) by keeping the group I genes as a seed, added randomly pick up genes (100 times) which were not miRNA targets, and repeated the analysis for each set. The reduced abundance of mRNAs targeted by the 36 up-regulated miRNAs remained significant for each random gene set (RR < 1, *P *< 0.05), suggesting that the targets of these miRNAs make a strong contribution to the low expression of general miRNA targets.

**Figure 4:**
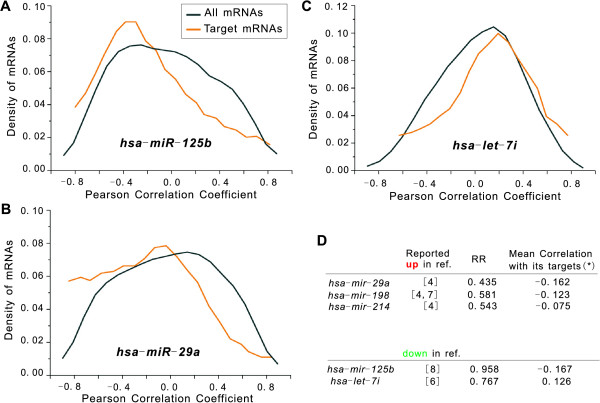
**Relationship between the transcript levels of individual miRNAs and their targets in prostate cancer**. Global density distribution of the Pearson correlation coefficients between the expression of mRNAs and the expression of *miR-125b *(A), *miR-29a *(B) and *let-7i *(C). The black-lined curves show the distribution of the correlation coefficients for all mRNAs and the orange-lined curves show the correlation coefficient distribution for only PicTar target mRNAs of *miR-125b*, *miR-29a *and *let-7i*. (D) A list of differential expressed miRNAs (ref: reference) that not only show distinct mean correlation coefficients between with their targets and with all mRNAs (asterisk denotes *P *< 0.05) but also have concordant RR values.

To further investigate the relationship between the abundance of individual miRNAs and their targets in prostate cancer, we performed a correlation analysis between the transcript levels of individual miRNAs and those of their putative targets using a dataset containing the expression measurements of both miRNAs and mRNAs in ten prostate tumours and ten corresponding surrounding non-tumour tissues [19]. Of the 137 studied miRNAs with more than 50 targets found in the mRNA expression profiles, 67% (92) showed negative mean Pearson correlation coefficients with their targets, 18% (24, including *miR-125b*, *miR-29a*, and *miR-194*) and 7% (9, including *let-7i*, and *miR-138*) showed significantly stronger negative and positive, respectively, mean correlations with their targets than with all mRNAs (*P *< 0.05) [see Additional file 3]. These results suggest that while binding of miRNAs to their target sequences may largely lead to the reduction of the transcript levels of target mRNAs, it may sometimes lead to mRNA sequestration and cellular accumulation of the inhibited mRNAs in prostate tumours [19]. We also determined the global distribution of the Pearson correlation coefficient between each miRNA of interest and either all mRNAs or the putative targets of the miRNA. For three miRNAs, *miR-125b*, *miR-29a *and *let-7i*, the distribution of the correlation coefficients was notably different between all mRNAs and the miRNA-target mRNAs (Figure 4A, B, C). The density distribution curves for targets of *miR-125b *and *miR-29a *extended toward negative correlation coefficients, indicating that the transcript levels of some target mRNAs may be reduced by *miR-125b *and *miR-29a*. On the contrary, the distribution curves for targets of *let-7i *extended toward positive correlation coefficients, indicating that the overall effect of binding of *let-7i *to its target sequence in prostate tumours may be mRNA sequestration. A list of differentially expressed miRNAs (in prostate cancer) that not only showed distinct mean correlations between with their targets and with all mRNAs but also had concordant RR values is shown in Figure 4D. For example, *miR-29a*, which was reported to be up-regulated in prostate tumour and showed strong negative mean correlation with its targets, had a very low RR value (0.435). *let-7i*, which was reported to be down-regulated and showed strong positive mean correlation with its targets, also had a low RR value (0.767). *miR-125b*, which was reported to be down-regulated and showed strong negative mean correlation with its targets, had a relatively high RR value (0.958). These miRNAs may be of special interest in future prostate cancer research.

### Positive correlation between the reduced abundance of miRNA targets and the number of target-site types in their 3' UTRs in prostate cancer

MiRNA target predictions rely strongly on the sequence characteristics of 3' UTRs, which have known functions in the stability, localisation, and translation of mRNA [20]. Most miRNA targets contain more than one type of target site in their 3' UTRs, implying that stringent regulation by one miRNA is rare. For example, the target mRNAs of the 36 up-regulated miRNAs included a major fraction of all PicTar targets (5514 of 8177) which were shared by 168 miRNAs. Interestingly, we found that the average number of target-site types in the target mRNAs of up-regulated miRNAs (≈ 10) was significantly larger than the average number found in the target mRNAs of all other miRNAs (≈ 2, *P *< 10^-100^, Wilcoxon-Man-Whitney test).

**Figure 5:**
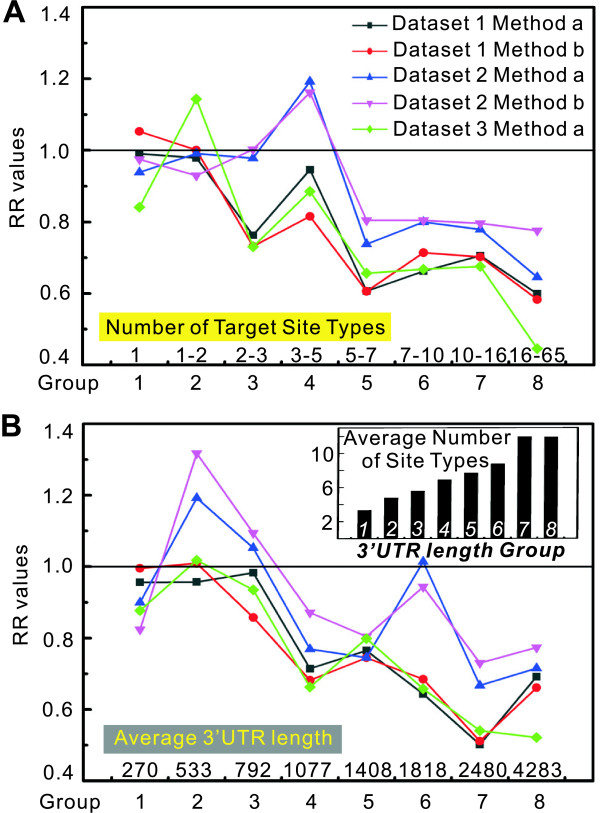
**Correlation between the reduced abundance and the number of target-site types**. PicTar targets were divided into eight groups according to the number of target-site types or the length of 3' UTRs. (A) A positive correlation between the reduced abundance of miRNA targets and the number of target-site types the target mRNAs contain. (B) A positive correlation between the propensity for low expression and the length of 3' UTRs of miRNA-target mRNAs. Average number of miRNA target-site types in each group divided by 3' UTR length is shown above.

It has been suggested that multiple miRNAs may act together to regulate a target mRNA [9]. However, it is still unclear if different miRNAs act *in vivo *in any kind of synergistic style. To further study the relationship between the reduced abundance of miRNA targets and the regulatory complexity of miRNAs (the number of miRNA target-site types in the 3' UTRs of the targets) in prostate cancer, we divided the PicTar targets into eight groups (≈ 1022 miRNA targets per group), according to the number of miRNA target-site types the mRNAs contain, and calculated the RR value for each group. Interestingly, a significant positive correlation was found between the propensity for low expression and the number of miRNA target-site types in each group (Spearman's rank correlation, R_s _> 0.7, *P *< 0.05, N = 8). As shown in Figure 5A, the propensity for low expression of the target groups increased with the number of target-site types.

We found that the down-regulation of miRNA targets in tumour extracts correlated with the length of the 3' UTRs of these mRNAs (Figure 5B). It is not surprising to find more sites just by chance if the 3' UTR is longer. To ask whether this positive correlation is a side effect of the variations of 3' UTR length, we divided the PicTar targets into eight groups according to the length of their 3' UTRs and calculated the average number of target-site types for each group. Since genes with more miRNA sites would have not only relatively longer 3' UTRs but also significantly higher site densities (sites/kb) of 3' UTR sequence [12], the longer 3' UTRs does not always contain more target sites. This was seen in Figure 5B, where group 7 and 8 contained a similar average number of target-site types though they have distinct average 3' UTR length. If the reduced abundance of miRNA targets simply results from the variation of 3' UTR length, one would expect that the group with longest 3' UTRs would show the lowest RR value. However, group 7, which contained the most target-site types, but not the longest 3' UTRs, showed the lowest RR value for most datasets (all except dataset 3, which contained a relatively small number of mRNAs for calculation). Therefore, the positive correlation cannot simply be explained by variations in 3' UTR length, but is more likely due to the increasing number of target-site types.

It is well known that cancer cells epigenetically silence a number of genes by CpG island methylation. We next asked if the global down-regulation of miRNA targets in prostate cancer was a side effect of gene silencing by CpG island methylation. We found that the miRNA targets had significantly more CpG islands (average: 1.55) than all mRNAs (average: 1.38, *P *< 0.01, Wilcoxon-Man-Whitney test). However, randomly chosen groups of non-miRNA-target mRNAs with the same (or more) number of CpG islands as the targets, did not exhibit the same global down-regulation as the miRNA targets (RR > 1, *P *> 0.05). Moreover, we did not find correlations between the 3' UTR length (or miRNA target-site types) and the number of CpG islands (*P *> 0.05, spearman correlation test). Thus, the global down-regulation of miRNA targets cannot simply be explained by gene silencing due to CpG islands methylation.

### Reduced transcript levels of miRNA targets in androgen-independent prostate cancer when compared with androgen-dependent prostate cancer

**Figure 6:**
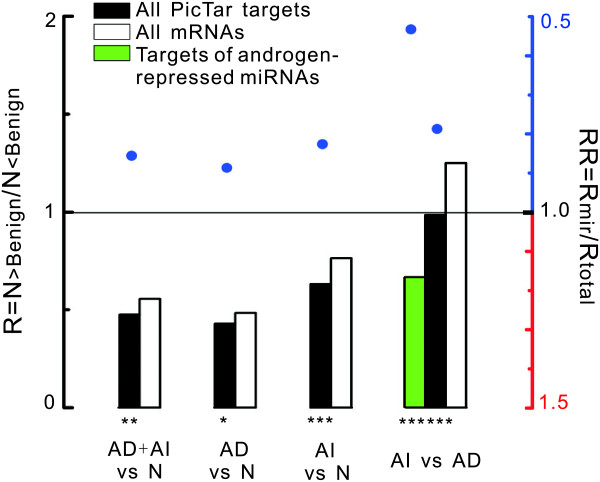
**Transcript levels of miRNA targets in androgen-independent and androgen-dependent prostate cancer**. The transcript levels of all miRNA targets in androgen-dependent (AD) prostate cancer, androgen-independent (AI) prostate cancer and normal prostate tissues (N) were compared with each other. The transcript level of the targets of androgen repressed miRNAs in AI prostate cancer was also investigated. Black and white bars represent R values of all miRNA targets and total mRNAs, respectively. Green bar represents R value of PicTar target mRNAs of androgen-repressed miRNAs. RR values and *P *values are analyzed as in Figure 1A.

The main treatment for prostate cancer is androgen ablation or chemical castration. Despite the general success of anti-androgen therapy, a negative outcome of this treatment is the appearance of androgen-refractory tumours with an eventually fatal prognosis. Thus, understanding the molecular mechanisms of the transition of prostate cancers from androgen dependence to independence remains an important challenge. In this study, we investigated the expression levels of miRNA targets in androgen-dependent (AD) prostate cancer and androgen-independent (AI) prostate cancer using a dataset previously published by our group [21]. As shown in Figure 6, the miRNA target genes showed lower transcript abundance in all prostate cancer (AD+AI), AI or AD prostate cancer than in normal prostate tissue (RR < 1, *P *< 0.05). Intriguingly, we found that the transcript levels of miRNA targets were significantly lower in AI prostate cancer than in AD prostate cancer (RR < 1, *P *< 0.05). Since some miRNAs are androgen regulated, we further investigated the transcript levels of the target group of androgen-repressed miRNAs (miRNAs that were down-regulated after androgen treatment in androgen-sensitive LNCaP cells) reported by S. Ambs et al. [19]. Using the aforementioned method, we divided all PicTar targets into two groups. Group I contained 1012 targets of 6 androgen-repressed miRNAs (5 of the 6 had conserved PicTar targets). Group II contained all other predicted miRNA targets (7165 targets). We found that the predicted targets of the 5 androgen-repressed miRNAs were significantly more likely to be down-regulated in AI prostate cancer (when compared with AD prostate cancer) than the predicted targets of all other miRNAs (*P *= 0.01). This result suggests that these androgen-repressed miRNAs may have an important influence on the expression of their targets in AI prostate cancer. Further, they may play an unknown function in the transition of prostate cancers from androgen dependence to independence.

### Down-regulation of miRNA targets in other cancer types and predicted function of these protein-coding genes in prostate cancer

To assess the basic function of down-regulated miRNA targets in prostate cancer, we used GO term analysis to identify the overrepresented biological processes in the group of down-regulated miRNA targets (referred to as "targets"), as well as the other reduced mRNAs that lacked conserved miRNA sites (referred to as "antitargets"). We used the mRNA list from dataset 1 (Method b, Medium cut-off), which contained a relatively large number of altered mRNAs, for this analysis. As shown in Table 2, the enrichment level in the targets was much higher than in the antitargets in most categories, especially "regulation of biological process" (adjusted enrichment *P *value = 7E-67 for targets versus 2E-08 for antitargets) and "multicellular organismal development" (adjusted enrichment *P *value = 4E-83 for targets versus 4E-19 for antitargets). For a more in-depth pathway analysis, we performed a KEGG pathway database query using the down-regulated targets and the antitargets (Table 3). At an adjusted *P *value < 0.05, 9 pathways were found to be enriched in the down-regulated miRNA targets, such as "regulation of action cytoskeleton", "Wnt signalling pathway", "Focal adhesion", "MAPK signalling pathway" and "ECM-receptor interaction". These dysregulated pathways are implicated in cell motility, cell proliferation, cell differentiation, cell migration and signal transduction. In contrast, the antitargets were not found to be enriched in any pathway. Since the low expression pattern observed here is largely seen in the targets containing multiple target-site types, our data may suggest that down-regulated targets in prostate cancer consist mostly of key cellular regulators and such regulators are themselves highly regulated at multiple levels, including regulation by miRNAs, which may result in the coordinate repression of the target mRNAs involved in regulatory systems and developmental processes.

**Table 2:** Biological processes of down-regulated miRNA targets and antitargets in prostate cancer

		Targets (1296)		AntiTargets (1293)	
		
GO Term	Biological Process	mRNAs	*P *(over)^†^	mRNAs	*P *(over)^†^
GO:0009987	Cellular process	1000	**1.E-47**	874	1.E-11
GO:0008152	Metabolic process	627	2.E-04	627	2.E-04
GO:0043170	Macromolecule metabolic process	545	4.E-12	473	2.E-02
GO:0050789	Regulation of biological process	494	**7.E-67**	317	2.E-08
GO:0007154	Cell communication	405	**2.E-39**	254	2.E-02
GO:0007275	Multicellular organismal development	302	**4.E-83**	180	4.E-19
GO:0030154	Cell differentiation	202	**4.E-43**	133	4.E-12
GO:0008219	Cell death	107	**4.E-24**	67	4.E-06

^†^: Over-represent *P *value was calculated using Hyper-Geometric (HG) distribution and adjusted. *P *values < 1E-20 (for mRNAs overrepresentation in each category) are in bold.

**Table 3:** KEGG pathway analysis of down-regulated miRNA targets and antitargets in prostate cancer

		Targets (316^†^)		AntiTargets (434^†^)	
		
KEGG Term	Pathway	InMap^‡^	*P *value^§^	InMap^‡^	*P *value^§^
hsa04810	Regulation of actin cytoskeleton	37	**6.25E-06**	23	0.86
hsa04360	Axon guidance	25	**7.96E-05**	9	1
hsa04310	Wnt signaling pathway	26	**2.98E-04**	9	1
hsa04510	Focal adhesion	31	**3.13E-04**	32	0.11
hsa04010	MAPK signaling pathway	36	**1.05E-03**	24	0.96
hsa04512	ECM-receptor interaction	17	**1.64E-03**	14	0.32
hsa04916	Melanogenesis	16	**3.20E-02**	7	0.84
hsa04070	Phosphatidylinositol signaling system	13	**4.12E-02**	7	0.96
hsa05130	Pathogenic Escherichia coli infection	10	**3.98E-02**	5	0.98

^†^: The total number of targets or antitargets found in KEGG pathway database. ^‡^: The number of targets or antitargets found in each pathway. ^§^: Adjusted *P *values < 0.05 are in bold.

To determine whether the global down-regulation of miRNA targets is common in human cancers, we extended the global analysis to four other prevalent cancers, including three solid tumours and one leukaemia (Table 1). Significant differences in transcript levels between miRNA targets and total mRNAs were observed for all four cancers [see Additional file 1]. Strikingly, the expression levels of miRNA targets were significantly lower in breast cancer, lung adenocarcinoma and acute myeloid leukaemia than in corresponding normal tissues (RR < 1, *P *< 0.05). In contrast, the comparison of hepatocellular carcinoma and non-tumour liver tissue yielded an opposite relationship between the abundance of miRNA target and total mRNAs (RR > 1, *P *< 0.05). In this analysis, an RR value greater than 1 indicated an increased abundance of miRNA targets when compared with all mRNAs. The globally increased abundance of miRNA targets may reflect different mechanisms of regulation in liver cancer. Our extended analysis indicates that the differences in transcript levels between miRNA targets and total mRNAs may be a common feature in human cancers.

## Discussion

Different expression levels between miRNA targets and total mRNAs have been uncovered in the comparison between mature tissues and embryos [22] and miRNAs have been suggested to confer precision and robustness to developmental processes. In this study, we initially reported that miRNA targets expressed less on a global scale than total mRNAs in prostate tumours, relative to benign prostate tissues. Analysis of the protein levels of miRNA targets suggests that the level of protein expression of miRNA targets may also be reduced, in agreement with a recent study which reported that the regulation of protein-coding genes by miRNAs was quite similar on the transcript and protein levels [14]. Moreover, our data showed that the transcript abundance of the targets of androgen-repressed miRNAs was significantly lower than the abundance of the targets of all other miRNAs in androgen-independent prostate cancer. The abnormal expression pattern of miRNA targets was also seen in three other cancer types, suggesting that it may be a common feature of the human cancer transcriptome.

We also found a trend for an increased down-regulation of mRNAs with longer 3' UTRs and more target-site types, consistent with a recent study showing that proliferating cells express mRNAs with shortened 3' UTRs and fewer miRNA target sites [23]. It has been reported that for proteins with more interacting partners, their genes tend to be regulated by more miRNA types [24,25]. Genes with more interactions may require more elaborate regulation at the posttranscriptional level because unwanted output of these proteins may lead to a more severe fitness effect. Moreover, miRNAs have been proposed to primarily target downstream network components such as transcription factors [26]. Disrupted expression of the highly regulated miRNA target genes may reflect the fact that the regulatory network in cancer cells departs from the normal regulatory routine presented in benign cells. The molecular mechanisms determining the intriguing expression patterns of miRNA targets in cancer cells presented in this study remain to be elucidated. Based on our analysis, there are three potential reasons discussed below.

First, the abnormal transcript abundance of miRNA targets may indicate a significant influence of miRNAs on the expression of their target genes in prostate tumours. This view is supported by three observations: (1) the targets of 36 up-regulated miRNAs made a strong contribution to the low expression of all miRNA targets; (2) there was a weak (but significant) negative correlation between the score of the differential expression of individual miRNAs (published by Volinia et al.) and the RR value of the miRNA's targets; and (3) the propensity for low expression increased with the number of target-site types embedded in the 3' UTRs of the miRNA targets, suggesting the possibility of synergistic regulation by multiple different miRNAs in prostate cancer. In general, lower transcript level is attributed to transcription inhibition or mRNA decay. Since miRNA target prediction relies strongly on the characteristics of 3' UTRs, translational control by 3' UTRs may play a role in the down-regulation of miRNA targets in prostate cancer. It has been demonstrated that miRNAs can promote rapid mRNA degradation by accelerating deadenylation [1] and that miRNAs are involved in AU-rich Element (ARE)-mediated mRNA instability [27]. Therefore, the low expression of miRNA targets may result from the action of miRNA-mediated mRNA decay in prostate cancer. Up-regulation of miRNAs in prostate tumours is common [3,4,19] and is consistent with the known oncogenic activity of many miRNAs [28,29]. It has been reported that *Dicer *and other genes involved in miRNA processing are up-regulated in prostate cancer [19], indicating that the prostate tumour is more efficient than normal prostate tissue at processing miRNA precursors into mature miRNAs. These observations support the idea that miRNAs may be up-regulated on a global scale in prostate cancer, consistent with the global down-regulation of their targets.

It should be noted that the global down-regulation of miRNA targets is an overall effect that does not negate the fact that some miRNA targets are up-regulated in prostate tumours. A recent miRNA profiling study showed a tumour gene signature that contains up-regulated and down-regulated miRNAs in prostate cancer [19]. This study also showed that binding of miRNAs to 3' UTR sequences can lead to both degradation and accumulation of the targeted mRNA in cancer cells. In the correlation analysis between the expression level of individual miRNAs and the expression level of their putative targets, we confirmed this observation on a global scale. More specifically, both an inverse and a positive correlation could occur between a miRNA and its target mRNAs in prostate cancer cells. Since a miRNA can regulate multiple targets and a single mRNA can be targeted by several different miRNAs, the global down-regulation of miRNA targets may largely depend on the overall effect of miRNA regulation. The second potential reason is that the global down-regulation of miRNA targets is an overall effect that may depend on: (1) the reduction of mRNA expression that may be caused by the up-regulated miRNAs (such as *miR-29a*); (2) the decrease of mRNA sequestration that may be caused by the down-regulated miRNAs (such as *let-7i*); and (3) the moderate up-regulation of some targets of the down-regulated miRNAs (such as *miR-125b*). Furthermore, this potential reason can explain the fact that global up-regulation of miRNA targets was observed for hepatocellular carcinoma, a tissue which has roughly equal numbers of up and down-regulated miRNAs [30].

The third reason is related to a perplexing problem: several other miRNA profiling studies showed widespread down-regulation of miRNAs in prostate cancer [5-8], and some of the miRNAs reported to be up-regulated by Volinia et al. overlapped with some of the miRNAs reported to be down-regulated by another profiling study [7]. If most miRNAs are truly down-regulated in prostate cancer, the global down-regulation of miRNA targets may not be causatively linked to the expression levels of the miRNAs themselves. As miRNA targets have relatively long 3' UTRs (with known functions in the stability, localisation, and translation of mRNA) and more CpG islands (which may be methylated in cancers) than non-targets, a third explanation is that the target genes that are down-regulated in prostate cancer are key cellular regulators and such key regulators are themselves highly regulated at multiple levels (transcriptionally and post-transcriptionally), including regulation by miRNAs.

There are several explanations for the discrepancies in miRNA profiling studies. First, Volinia et al. [4] and Ambs et al. [19] used total RNA while other studies [5-8] used purified small RNA samples (from 18 to 300 nt). Purification might introduce errors into miRNA expression comparisons. For example, there is presently no way to judge the different proportions of miRNAs within the pool of total RNAs [31]. If cancer and benign tissue have different proportions of miRNA content, the validity of the analysis based on the fundamental assumption that same amount of miRNAs is extracted from the same amount of total RNAs is thrown into doubt. Second, it has been suggested that purification of the small RNA fraction could reduce nonspecific hybridisation to longer miRNA precursors. If there is a block in precursor miRNA processing in prostate cancer without a corresponding decrease in transcription, this could result in the inconsistency. A third explanation would be the differences in samples number. Volinia et al. and Ambs et al. analysed > 50 prostate cancer samples while other studies did not reach this size. Cancer is a heterogeneous disease, and the heterogeneity of tumour samples might contribute substantially to the results. It is not surprising that miRNA expression profiles published by different researchers are inconsistent, because miRNA profiling technology is still in its infancy. For example, researchers generally adopt "tried-and-true" methodologies from cDNA microarray technology for miRNA expression analysis, but the relatively small number of probes on miRNA microarrays may render these high-density approaches ineffective.

As to the basis of our investigation, the gene expression microarray and miRNA target prediction data have proved to be useful for gaining biological knowledge [13,22,24-26]. Although these datasets are far from being complete and may contain noise, it is unlikely that these flaws could totally distort the results. Since consistent results were seen across various datasets generated by independent groups, the noise of microarray data and false positives in miRNA target predictions appear to have no serious effects on our study. Furthermore, the overall significances inferred from thousands of mRNAs would be strong enough to reflect real biology. The strength of our global analysis lies in the noise reduction effect, as well as the identification of general trends of miRNA target expression that would not have been discovered by individual investigation of single miRNA targets. Cancer is an extremely complex and heterogeneous disease [32]. It should be noted that our data did not conclusively distinguish among the three possible mechanisms discussed above, and the detailed molecular mechanisms responsible for the abnormal expression of miRNA targets remain to be thoroughly elucidated. Future experiments or large microarray studies are needed to clarify the possible mechanisms.

## Conclusion

In conclusion, our global gene expression analysis, along with further investigations, suggests that miRNA targets have significantly reduced transcript abundance in prostate cancer, when compared with the combined pool of total mRNAs. The abnormal expression patterns of miRNA targets could be a common feature of the human cancer transcriptome. These observations raise the possibility that miRNA may have global functions in human prostate cancer. Our study may help to shed new light on miRNA functions in cancer transcriptomics, when unprecedented opportunities to study the regulatory control mediated by miRNAs are given by the accumulation of cDNA, miRNA expression and proteomic datasets.

## Methods

### Data collection

The gene expression microarray datasets used in this study are listed in Table 1, including five datasets in human prostate cancer and four in other cancer types (breast cancer, lung adenocarcinoma, acute myeloid leukaemia and liver cancer) [33-36]. We also used one immunobloting proteomic dataset in prostate cancer. Gene expression datasets were of two general types, two channel ratio data (cDNA datasets) and single channel intensity data (Affymetrix datasets), and were generally given in a single matrix file format. All gene expression datasets were normalized by the authors of these studies. Probe IDs were converted to RefSeq mRNA IDs using ID converter [37], if necessary. We used two complete lists of human miRNA targets published by Lewis et al. and Krek et al. These miRNA target prediction datasets were downloaded from the most recently updated websites.

### Determining the mRNA groups with high and low expression in cancers relative to normal tissues

We reviewed the samples profiled for each of the gene expression datasets and chose the samples of classes of interest for further analysis. For each of the two classes e.g. cancer versus normal, the probe sets with absent calls (Affymetrix) or missing values (cDNA) in excess of 50% of the samples were filtered out. For each probe, we first averaged across sample replicates, then directly compared the median expression values between the two classes (cancer and normal). After that, we averaged the ratios (fold changes) for probes of the same RefSeq transcripts in each dataset and determined whether a mRNA had a high or low expression level (Method a). In dataset 1 and 2, where the expression measurements of matched samples (prostate cancers and benign prostate samples from same patients) were provided, another method (Method b) was used to rule out the bias of single method. We determined if a mRNA altered for each patient by computing the expression ratios of each patient, and only those genes showing alterations in most (based on different cut-offs of median ratio of paired samples) patients were considered to be altered. In dataset 2, only the "well-measured genes" defined by the author were included in our analysis. Moreover, in order to rule out the bias of single cut-off (or threshold) for identifying mRNAs with low and high expression, we also chose three different cut-off values for each comparison (High, Medium and Low [see Additional file 1]). For datasets 5–7 of three other cancer types, we directly downloaded the over- and under-expression gene lists from Oncomine database [38].

### Calculation of R and RR values and resampling statistical tests

Here we modified a method from Yu et al. 2007 [22]. After obtaining the mRNAs with high and low expression in cancers relative to normal tissues, we counted the number of miRNA targets that fell in the high expression group (N_ > Benign_) and divided it by the amount of targets in the low expression group (N_ < Benign_). The obtained ratio was named as R_mir _(R_mir _= N_ > Benign_/N_ < Benign_). As a control, the same calculations for all mRNAs were undertaken and the ratio R_total _was obtained. The ratio of R_mir _to R_total _was computed and termed as RR (RR = R_mir_/R_total_). The RR value is a surrogate for an increased or decreased abundance of miRNA targets relative to total mRNAs. An RR value less than 1 indicates that the targets prefer a lower expression. We undertook a resampling test to judge the statistical significance of the global observation. In each test, we randomly picked the same number of mRNAs as the number of miRNA targets from total altered mRNAs. We then calculated the ratio of high expression mRNAs to low expression mRNAs in this random sub-pool and termed it as R_random_. The randomization tests were performed 5000 times and the number of times (n) was counted when R_random _> R_mir_. *P*-value was defined as n/5000. If *P*-value < 0.05, we determined the expression level of miRNA targets was significantly lower than that of total mRNAs. We used two miRNA target predictions for this analysis and performed correlation analysis of the RR values obtained from each of the two target predictions.

### Correlation analysis between the expression levels of individual miRNAs and those of their putative targets

For the dataset containing expression measurements of both miRNAs and mRNAs in ten prostate tumours and ten corresponding surrounding non-tumour tissues [19], we calculated the log expression ratios of paired samples for each miRNA and each mRNA. Then the Pearson correlation coefficients between each miRNA and each mRNA were calculated using the expression ratios. We also determined the global density distribution of the Pearson correlation coefficients between the miRNA of interest and either all mRNAs or the predicted targets of the miRNA.

### Analysis of the relationship between the RR values and 3' UTR length and the number of predicted miRNA target-site types

The number of target-site types of each PicTar target mRNA was calculated. Since the underlying distributions of the numbers of miRNA target site types and RR values were not normal, Spearman's rank correlation test was used to determine the relations between these two variables. We extracted information of 3' UTR length of each mRNA from the UTResource database [39]. The human CpG islands data were extracted from Human CpG Island database [40].

### GO term analysis and KEGG pathway analysis

To study the function of down-regulated miRNA targets, we used the GO Term Mapper Web server [41]. We used default GOA slim file (a list of general GO terms) for annotating the down-regulated mRNAs. The KEGG database [42] batch entry allowed us to evaluate the large set of down-regulated miRNA targets and antitargets. In order to evaluate the enrichment level of mRNAs to a specific biological process or pathway, we used the Hyper-Geometric (HG) distribution to calculate the enrichment *P *value. A more detailed explanation of this distribution was previously described [43].

## Authors' contributions

RS planed and designed the study, performed the data analysis and the further analysis in bioinformatics, wrote the main draft of the paper, and generated the figures. XF performed the data analysis and organized this research. YL, YX and YM organized all the research and provided advice for preparing the manuscript. All authors read and approved the final manuscript.

## Supplementary Material

Additional file 1 **Supplementary Table 1**. Comparison of miRNA target expression between in cancers and in normal tissues. The complete dataset of comparing the expression levels of miRNA targets between in cancers and in normal tissues. Click here for file

Additional file 2 **Supplementary Table 2**. A weak negative correlation between the differential expression scores of individual miRNAs and the RR values of their targets. The differential expression scores of individual miRNAs and the RR values of their targets. Click here for file

Additional file 3 **Supplementary Table 3**. Correlation analysis between the transcript levels of individual miRNAs and those of their putative targets using a dataset containing the expression values of both miRNAs and mRNAs in ten prostate tumours and ten corresponding surrounding non-tumour tissues. The average Pearson correlation coefficients for individual miRNAs with either target mRNAs or all mRNAs. Click here for file
