# Supplementary material for: Global gene expression analysis reveals reduced abundance of putative microRNA targets in human prostate tumours
Source: BMC Genomics. 2009 Feb 26;10:93. doi: 10.1186/1471-2164-10-93 (PMC2653538; doi:10.1186/1471-2164-10-93)
Supplement: Additional file 1 — Supplementary Table 1. Comparison of miRNA target expression between in cancers and in normal tissues. The complete dataset of comparing the expression levels of miRNA targets between in cancers and in normal tissues. [file 1471-2164-10-93-S1.pdf]

Supplementary Table 1: Comparison of miRNA target expression between in cancers and in normal tissues

L: Localized prostate cancer
M: Metastatic prostate cancer
B: Benign prostate tissue

Prostate Cancer

Number of Total expressed mRNAs (L vs B)

DataSet	Cut-off	>B	≈B	<B	total mRNAs	R_{total} (>B/<B)
	L(1.05 1/1.05)	5414	4838	4542	14794	1.191985909
1 (Method a)	M(1.1 1/1.1)	3425	8387	2982	14794	1.148558015
	H(1.2 1/1.2)	1269	12100	1425	14794	0.890526316
	L(1.05 1/1.05)	7172	1479	6142	14793	1.167697818
1 (Method b)	M(1.2 1/1.2)	5138	5333	4322	14793	1.188801481
	H(1.5 1/1.5)	2269	10397	2127	14793	1.066760696
	L(0.05 -0.05)	1388	311	1595	3294	0.870219436
2 (Method a)	M(0.1 -0.1)	1205	652	1437	3294	0.83855254
	H(0.2 -0.2)	873	1280	1141	3294	0.765118317
	L(1.05 1/1.05)	1486	53	1755	3294	0.846723647
2 (Method b)	M(1.2 1/1.2)	1272	496	1526	3294	0.833551769
	H(1.6 1/1.6)	905	1218	1171	3294	0.772843723
	L(1.05 1/1.05)	1857	1170	1362	4389	1.363436123
3 (Method a)	M(1.1 1/1.1)	1221	2193	975	4389	1.252307692
	H(1.2 1/1.2)	468	3395	526	4389	0.88973384

Number of Total expressed mRNAs (L+M vs B)

DataSet	Cut-off	>B	≈B	<B	total mRNAs	R_{total} (>B/<B)
1 (Method a)	M(1.1 1/1.1)	4006	7107	3681	14794	1.088291225
2 (Method a)	M(0.1 -0.1)	1187	620	1487	3294	0.798251513
3 (Method a)	M(1.1 1/1.1)	1498	1786	1105	4389	1.355656109

Number of Total expressed mRNAs (M vs B)

DataSet	Cut-off	>B	≈B	<B	total mRNAs	R_{total} (>B/<B)
1 (Method a)	M(1.1 1/1.1)	5593	3472	5729	14794	0.976261128
2 (Method a)	M(0.1 -0.1)	1103	172	2019	3294	0.546310054
3 (Method a)	M(1.1 1/1.1)	1808	1179	1402	4389	1.289586305

Number of Total expressed mRNAs (M vs L)

DataSet	Cut-off	>L	≈L	<L	total mRNAs	R_{total} (>L/<L)
1 (Method a)	M(1.1 1/1.1)	5053	3964	5777	14794	0.874675437
2 (Method a)	M(0.1 -0.1)	989	295	2010	3294	0.492039801
3 (Method a)	M(1.1 1/1.1)	1511	1622	1256	4389	1.203025478

Number of Total expressed mRNAs (B1 vs B2)

DataSet	Cut-off	>B1	≈B1	<B1	total mRNAs	R_{total} (>B1/<B1)
1 (Method a)	M(1.1 1/1.1)	3445	7978	3371	14794	1.021951943

Let's call: C_a = average expression value of cancer samples
B_a = average expression value of benign samples
then: Cutof (1.1 1/1.1) means $C_a/B_a > 1.1$ or $C_a/B_a < 1/1.1$
Let's call: C_{al} = average log expression value of cancer samples
B_{al} = average log expression value of benign samples
then: Cutof (0.1 -0.1) means $C_{al}-B_{al} > 0.1$ or $C_{al}-B_{al} < -0.1$

Cut-offs were determined or regulated to yeild appropriate number of mRNAs in each group

Prostate Cancer

PicTar

Number of expressed miRNA Targets (L vs B)

DataSet	Cut-off	>B	≈B	<B	total targets	R_{mir} (>B/<B)	*P-value*	RR
	L(1.05 1/1.05)	1574	1618	1673	4865	0.940824866	<.0002	0.78929
1 (Method a)	M(1.1 1/1.1)	984	2755	1126	4865	0.873889876	<.0002	0.76086
	H(1.2 1/1.2)	339	3993	533	4865	0.636022514	<.0002	0.71421
	L(1.05 1/1.05)	2147	454	2264	4865	0.948321555	<.0002	0.81213
1 (Method b)	M(1.2 1/1.2)	1512	1673	1680	4865	0.9	<.0002	0.75707
	H(1.5 1/1.5)	662	3313	890	4865	0.743820225	<.0002	0.69727
	L(0.05 -0.05)	546	126	728	1400	0.75	0.004	0.86185
2 (Method a)	M(0.1 -0.1)	472	268	660	1400	0.715151515	0.003	0.85284
	H(0.2 -0.2)	347	524	529	1400	0.655954631	0.01	0.85732
	L(1.05 1/1.05)	594	22	784	1400	0.757653061	0.02	0.89481
2 (Method b)	M(1.2 1/1.2)	506	208	686	1400	0.737609329	0.02	0.8849
	H(1.6 1/1.6)	363	496	541	1400	0.670979667	0.01	0.8682
	L(1.05 1/1.05)	642	508	648	1798	0.990740741	<.0002	0.72665
3 (Method a)	M(1.1 1/1.1)	426	896	476	1798	0.894957983	<.0002	0.71465
	H(1.2 1/1.2)	162	1376	260	1798	0.623076923	<.0002	0.7003

Number of expressed miRNA Targets (L+M vs B)

		>B	≈B	<B	total targets	R_{mir} (>B/<B)	*P-value*	RR
1 (Method a)	M(1.1 1/1.1)	1193	2322	1350	4865	0.883703704	<.0002	0.81201
2 (Method a)	M(0.1 -0.1)	461	248	691	1400	0.667149059	0.001	0.83576
3 (Method a)	M(1.1 1/1.1)	489	774	535	1798	0.914018692	<.0002	0.67423

Number of expressed miRNA Targets (M vs B)

		>B	≈B	<B	total targets	R_{mir} (>B/<B)	*P-value*	RR
1 (Method a)	M(1.1 1/1.1)	1761	1139	1965	4865	0.896183206	0.004	0.91797
2 (Method a)	M(0.1 -0.1)	401	75	924	1400	0.433982684	<.0002	0.79439
3 (Method a)	M(1.1 1/1.1)	624	511	663	1798	0.941176471	<.0002	0.72983

Number of expressed miRNA Targets (M vs L)

		>L	≈L	<L	total targets	R_{mir} (>L/<L)	*P-value*	RR
1 (Method a)	M(1.1 1/1.1)	1673	1280	1912	4865	0.875	0.5	1.00037
2 (Method a)	M(0.1 -0.1)	359	120	921	1400	0.389793702	<.0002	0.7922
3 (Method a)	M(1.1 1/1.1)	555	677	566	1798	0.980565371	<.0002	0.81508

Number of expressed miRNA Targets (B1 vs B2)

		>B1	≈B1	<B1	total targets	R_{mir} (>B1/<B1)	*P-value*	RR
1 (Method a)	M(1.1 1/1.1)	1116	2710	1039	4865	1.074109721	0.87	1.05104

RR = R_{mir}/R_{total}

RR<1

RR>1

P<0.05

P>0.05

Prostate Cancer

TargetScanS

Number of expressed miRNA Targets (L vs B)

DataSet	Cut-off	>B	≈B	<B	total targets	R_{mir} (>B/<B)	P-value	RR
	L(1.05 1/1.05)	1552	1604	1662	4818	0.933814681	<.0002	0.78341
1 (Method a)	M(1.1 1/1.1)	954	2742	1122	4818	0.85026738	<.0002	0.74029
	H(1.2 1/1.2)	325	3967	526	4818	0.617870722	<.0002	0.69383
	L(1.05 1/1.05)	2125	446	2247	4818	0.945705385	<.0002	0.80989
1 (Method b)	M(1.2 1/1.2)	1484	1675	1659	4818	0.894514768	<.0002	0.75245
	H(1.5 1/1.5)	644	3289	885	4818	0.727683616	<.0002	0.68214
	L(0.05 -0.05)	575	146	760	1481	0.756578947	0.006	0.86941
2 (Method a)	M(0.1 -0.1)	500	300	681	1481	0.734214391	0.009	0.87557
	H(0.2 -0.2)	363	568	550	1481	0.66	0.01	0.86261
	L(1.05 1/1.05)	632	23	826	1481	0.765133172	0.02	0.90364
2 (Method b)	M(1.2 1/1.2)	539	225	717	1481	0.751743375	0.03	0.90186
	H(1.6 1/1.6)	374	535	572	1481	0.653846154	0.004	0.84603
	L(1.05 1/1.05)	730	537	688	1955	1.061046512	<.0002	0.77822
3 (Method a)	M(1.1 1/1.1)	466	982	507	1955	0.91913215	<.0002	0.73395
	H(1.2 1/1.2)	178	1496	281	1955	0.633451957	<.0002	0.71196

Number of expressed miRNA Targets (L+M vs B)

DataSet	Cut-off	>B	≈B	<B	total targets	R_{mir} (>B/<B)	P-value	RR
1 (Method a)	M(1.1 1/1.1)	1195	2295	1328	4818	0.899849398	<.0002	0.82685
2 (Method a)	M(0.1 -0.1)	485	274	722	1481	0.671745152	0.001	0.84152
3 (Method a)	M(1.1 1/1.1)	547	833	575	1955	0.951304348	<.0002	0.70173

Number of expressed miRNA Targets (M vs B)

DataSet	Cut-off	>B	≈B	<B	total targets	R_{mir} (>B/<B)	P-value	RR
1 (Method a)	M(1.1 1/1.1)	1792	1090	1936	4818	0.925619835	0.05	0.94813
2 (Method a)	M(0.1 -0.1)	408	81	992	1481	0.411290323	<.0002	0.75285
3 (Method a)	M(1.1 1/1.1)	689	555	711	1955	0.969057665	<.0002	0.75145

Number of expressed miRNA Targets (M vs L)

DataSet	Cut-off	>L	≈L	<L	total targets	R_{mir} (>L/<L)	P-value	RR
1 (Method a)	M(1.1 1/1.1)	1691	1252	1875	4818	0.901866667	0.81	1.03109
2 (Method a)	M(0.1 -0.1)	371	132	978	1481	0.379345603	<.0002	0.77097
3 (Method a)	M(1.1 1/1.1)	597	745	613	1955	0.973898858	<.0002	0.80954

Number of expressed miRNA Targets (B1 vs B2)

DataSet	Cut-off	>B1	≈B1	<B1	total targets	R_{mir} (>B1/<B1)	*P-value*	RR
1 (Method a)	M(1.1 1/1.1)	1058	2724	1036	4818	1.021235521	0.49	0.9993

$RR = R_{mir}/R_{total}$

RR<1

RR>1

P<0.05

P>0.05

Prostate Cancer

Target enrichment in three groups

DataSet	Cut-off	*P* (Enrichment) PicTar >B	≈B	<B	*P* (Enrichment) TargetScanS >B	≈B	<B
	L	0.99	0.16	7.25E-12	0.99	0.14	2.64E-12
1 (Method a)	M	0.99	0.55	1.83E-10	0.99	0.36	3.57E-11
	H	0.99	0.27	8.61E-05	0.99	0.12	1.46E-04
	L	0.99	0.97	3.04E-18	0.99	0.98	1.15E-18
1 (Method b)	M	0.99	0.99	3.16E-23	0.99	0.98	3.83E-22
	H	0.99	0.99	5.39E-21	0.99	0.99	1.99E-21
	L	0.99	0.79	2.34E-04	0.99	0.25	1.49E-03
2 (Method a)	M	0.99	0.81	2.67E-04	0.99	0.29	7.54E-03
	H	0.98	0.93	6.35E-04	0.99	0.72	3.63E-03
	L	0.99	0.61	3.94E-03	0.99	0.64	5.24E-03
2 (Method b)	M	0.99	0.62	4.53E-03	0.99	0.44	0.016
	H	0.96	0.94	8.20E-04	0.99	0.83	4.98E-04
	L	0.99	0.02	1.59E-09	0.99	0.15	5.85E-08
3 (Method a)	M	0.99	0.57	1.14E-08	0.99	0.39	7.08E-08
	H	0.99	0.87	1.78E-05	0.99	0.89	8.27E-06

Other Cancer Types

C: Cancer **N: Normal**

Number of Total expressed mRNAs (C vs N)

DataSet	Cut-off	>N	≈N	<N	total mRNAs	R_{total} (>N/<N)
BC	M(0.1 -0.1)	4315	1845	4507	10667	0.957399601
LA		743		881	1624	0.843359818
AML	Oncomine	3308		3032	6340	1.091029024
LC		2873		2511	5384	1.144165671

PicTar

Number of expressed miRNA Targets (C vs N)

DataSet	Cut-off	>N	≈N	<N	total targets	R_{mir} (>N/<N)	*P* -value	RR
BC	M(0.1 -0.1)	1411	580	1743	3734	0.80952381	<.0002	0.84554
LA		284		404	688	0.702970297	0.03	0.83354
AML	Oncomine	983		1330	2313	0.739097744	<.0002	0.67743
LC		1113		943	2056	1.180275716	<.0002	1.03156

TargetScanS

Number of expressed miRNA Targets (C vs N)

DataSet	Cut-off	>N	≈N	<N	total targets	R_{mir} (>N/<N)	*P* -value	RR
BC	M(0.1 -0.1)	1426	586	1703	3715	0.83734586	0.003	0.8746
LA		279		418	697	0.667464115	0.002	0.79143
AML	Oncomine	1012		1462	2474	0.692202462	<.0002	0.63445
LC		1189		1033	2222	1.151016457	0.0008	1.00599

BC: Breast Cancer
LA: Lung Adenocarcinoma
AML: Acute Myeloid Leukemia
LC: Liver Cancer

RR = R_{mir}/R_{total}

RR<1
RR>1

P<0.05
P>0.05
