# Supplementary material for: Global gene expression analysis reveals reduced abundance of putative microRNA targets in human prostate tumours
Source: BMC Genomics. 2009 Feb 26;10:93. doi: 10.1186/1471-2164-10-93 (PMC2653538; doi:10.1186/1471-2164-10-93)

Supplementary Table 2

A weak negative correlation between the scores of differential expression of individual miRNAs (reported by Volinia S et al. 2006) and the RR values of their targets

miRNA Name	Score of differential expression (+: induced -: repressed)	RR value of target group (dataset 1, Method a, medium cutoff)
**hsa-miR-128a**	- 0.371	0.798850177
**hsa-let-7a**	-0.2817	0.767007299
**hsa-miR-218**	-0.227	0.858730127
**hsa-miR-63**	-0.0855	0.435328467
**hsa-miR-149**	-0.076	0.647411567
**hsa-miR-24**	-0.0075	1.335007299
**hsa-miR-199a-1**	0.0033	0.565927007
**hsa-miR-106a**	0.0064	0.688291355
**hsa-miR-27a**	0.0102	0.674759124
**hsa-miR-214**	0.0118	0.542747179
**hsa-miR-124a**	0.0134	0.700952617
**hsa-miR-26a**	0.0134	0.803683324
**hsa-miR-101**	0.0138	0.655553456
**hsa-miR-16**	0.0254	0.53473418
**hsa-miR-223**	0.0346	0.633205043
**hsa-miR-93**	0.0351	0.57384207
**hsa-miR-186**	0.0377	0.528613139
**hsa-miR-181b**	0.0441	0.599161761
**hsa-miR-29b**	0.0831	0.618410533
**hsa-miR-184**	0.086	0.652992701
**hsa-miR-206**	0.0934	0.611812981
**hsa-miR-32**	0.0982	0.684087591
**hsa-miR-211**	0.0988	0.599161761
**hsa-miR-146**	0.1021	0.497518248
**hsa-miR-92**	0.1081	0.664060373
**hsa-miR-17-5p**	0.1185	0.548445313
**hsa-miR-30c**	0.1195	0.760097324
**hsa-miR-199a-2**	0.1224	0.565927007
**hsa-miR-198**	0.1306	0.581383688
**hsa-let-7i**	0.1466	0.767007299
**hsa-miR-21**	0.152	1.160875912
**hsa-miR-191**	0.153	0.652992701
**hsa-miR-148**	0.1576	0.564750444
**hsa-miR-196**	0.1587	0.50669379
**hsa-miR-187**	0.1727	0.48665987
**hsa-miR-135**	0.178	0.478125308

**hsa-miR-25**	0.2094	0.580437956
**hsa-miR-29a**	0.2225	0.435328467
**hsa-miR-20a**	0.2609	0.456632214
**hsa-miR-34a**	0.2731	0.737844859
**hsa-miR-203**	0.3207	0.474903782
**hsa-miR-195**	0.3599	0.53473418
**hsa-let-7d**	0.4755	0.902317186

Spearman correlation coefficient = -0.342

P value = 0.025

N = 43

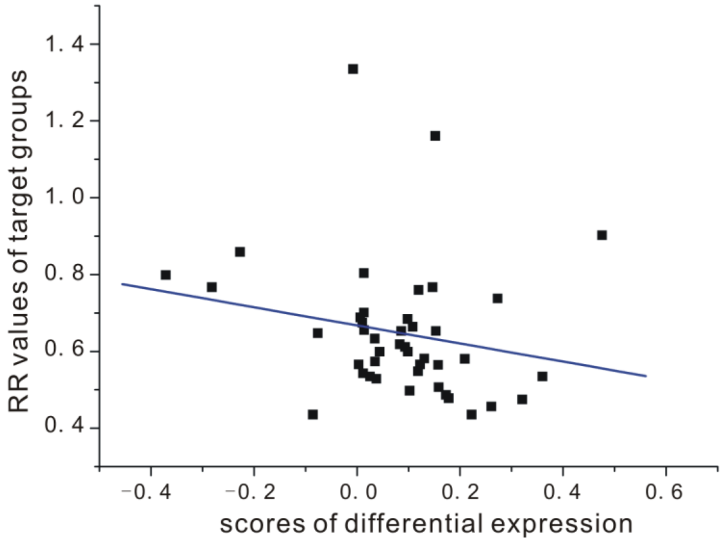

Supplement: Additional file 2 — Supplementary Table 2. A weak negative correlation between the differential expression scores of individual miRNAs and the RR values of their targets. The differential expression scores of individual miRNAs and the RR values of their targets. [file 1471-2164-10-93-S2.pdf]
