# Supplementary material for: Global gene expression analysis reveals reduced abundance of putative microRNA targets in human prostate tumours
Source: BMC Genomics. 2009 Feb 26;10:93. doi: 10.1186/1471-2164-10-93 (PMC2653538; doi:10.1186/1471-2164-10-93)
Supplement: Additional file 3 — Supplementary Table 3. Correlation analysis between the transcript levels of individual miRNAs and those of their putative targets using a dataset containing the expression values of both miRNAs and mRNAs in ten prostate tumours and ten corresponding surrounding non-tumour tissues. The average Pearson correlation coefficients for individual miRNAs with either target mRNAs or all mRNAs. [file 1471-2164-10-93-S3.pdf]

Supplementary Table 3

The correlation analysis between the expression levels of individual miRNAs and those of their putative targets using a dataset containing the expression values of both miRNAs and mRNAs in ten prostate tumors and ten corresponding surrounding nontumor tissues. Coloured rows list the miRNAs showing significant differences in average correlations between with target mRNAs (AC_T) and with all mRNAs (AC_A). Green denotes $AC_T < AC_A$ and red denotes $AC_T > AC_A$.

microRNA	Average pearson correlation coefficient Of target mRNAs （AC_T）	Average pearson correlation coefficient of all mRNAs (AC_A)	Adjusted P-value
hsa-miR-125b	-0.16728	-0.04871316	6.63E-07
hsa-let-7i	0.125556	0.037431352	1.76E-06
hsa-miR-29a	-0.16166	-0.016112524	4.60E-06
hsa-miR-200a	-0.10501	0.011979895	1.03E-05
hsa-miR-19b	-0.0442	0.028212989	1.41E-05
hsa-miR-9	-0.04393	0.027061322	1.75E-05
hsa-miR-138	0.064576	-0.016398742	0.001098
hsa-miR-205	-0.11461	-0.002973982	0.001743
hsa-miR-34b	0.117956	0.042537814	0.002147
hsa-miR-181c	0.045944	-0.004865924	0.007285
hsa-miR-125a	-0.09526	-0.039863029	0.008486
hsa-let-7f	-0.09286	-0.026437742	0.008855
hsa-miR-182	-0.00283	0.038732849	0.009832
hsa-miR-141	-0.02466	0.040363053	0.010257
hsa-miR-96	-0.08418	-0.038159617	0.012379
hsa-miR-137	-0.04647	0.005257203	0.013481
hsa-miR-16	-0.01688	0.020327987	0.014271
hsa-miR-214	-0.07513	-0.016500521	0.014881
hsa-miR-34c	-0.0739	-0.02246883	0.021019
hsa-miR-194	-0.11241	-0.043682557	0.027648
hsa-miR-150	0.073374	-0.003598796	0.027746
hsa-miR-20	-0.02916	0.008809955	0.029073
hsa-miR-217	-0.08928	-0.021600783	0.03037
hsa-miR-139	0.02583	-0.039000728	0.030536
hsa-miR-15b	-0.07138	-0.040253203	0.031581
hsa-let-7b	-0.10456	-0.052622956	0.032589
hsa-miR-200c	-0.01417	0.040164735	0.035129
hsa-miR-339	0.11598	0.004315971	0.035929
hsa-let-7a	-0.10068	-0.049673949	0.039115
hsa-miR-328	0.069286	0.005425835	0.039329
hsa-miR-134	0.077513	-0.060436276	0.03968

**hsa-miR-198**	-0.12269	-0.051835922	0.040331
**hsa-miR-98**	-0.02252	0.020455633	0.048573
**hsa-miR-195**	0.007762	0.044598043	0.050447
**hsa-miR-145**	-0.07276	-0.013875395	0.052785
**hsa-miR-301**	-0.07993	-0.045098176	0.056817
**hsa-miR-212**	-0.09542	-0.02510285	0.059845
**hsa-let-7d**	-0.07302	-0.023209458	0.060535
**hsa-miR-320**	-0.09341	-0.048429233	0.071266
**hsa-miR-188**	-0.04717	0.029101356	0.072616
**hsa-miR-302b**	-0.06514	-0.026755426	0.073187
**hsa-miR-302d**	0.015942	-0.020315039	0.074549
**hsa-miR-148a**	0.018748	0.063987257	0.079036
**hsa-miR-199b**	-0.09357	-0.036836421	0.081526
**hsa-miR-368**	-0.05684	0.007830441	0.083459
**hsa-miR-367**	0.049609	0.012327434	0.083905
**hsa-miR-101**	-0.05862	-0.023264335	0.098656
**hsa-miR-29c**	-0.02526	0.002751391	0.106169
**hsa-miR-142-3p**	-0.06149	-0.011742763	0.135593
**hsa-miR-15a**	0.001356	-0.025397342	0.1437
**hsa-miR-24**	-0.05957	-0.024281552	0.152052
**hsa-let-7c**	-0.04418	-0.014254904	0.174565
**hsa-miR-196a**	0.065238	0.01971221	0.183348
**hsa-miR-323**	0.060869	0.022957595	0.189876
**hsa-miR-186**	-0.00743	0.018482358	0.19666
**hsa-miR-144**	0.033893	0.063452337	0.207267
**hsa-miR-199a**	-0.04681	-0.010663584	0.209473
**hsa-miR-193**	-0.01205	-0.0493008	0.219639
**hsa-miR-28**	-0.08248	-0.035091391	0.22767
**hsa-miR-342**	-0.05029	-0.001214452	0.259512
**hsa-miR-29b**	0.062778	0.044011581	0.282401
**hsa-miR-32**	-0.02363	-0.005639408	0.284662
**hsa-miR-143**	-0.05863	-0.023198001	0.289314
**hsa-miR-128b**	-0.07784	-0.055720367	0.292586
**hsa-miR-10a**	-0.04311	-0.011358308	0.293941
**hsa-miR-30e**	-0.00929	-0.027970884	0.306121
**hsa-miR-107**	-0.02943	-0.012805135	0.336073
**hsa-miR-133b**	-0.04084	-0.020451652	0.359627
**hsa-miR-146**	-0.05771	-0.017718918	0.36746
**hsa-miR-10b**	-0.07732	-0.040773656	0.370622
**hsa-miR-33**	-0.01292	-0.048912267	0.384065
**hsa-miR-93**	0.001045	0.015060881	0.396325

**hsa-miR-17-5p**	-0.02551	-0.010228642	0.406684
**hsa-miR-129**	0.036351	0.01085992	0.433882
**hsa-miR-106b**	0.056568	0.04106796	0.442717
**hsa-miR-92**	-0.01908	-0.00461191	0.448906
**hsa-miR-155**	-0.05436	-0.033620463	0.451892
**hsa-miR-135a**	0.022537	0.007105572	0.465481
**hsa-miR-21**	-0.01549	0.014666078	0.467116
**hsa-let-7g**	-0.03378	-0.017972241	0.483347
**hsa-miR-183**	-0.01416	0.002779923	0.489101
**hsa-miR-103**	-0.03051	-0.01891831	0.499164
**hsa-miR-372**	0.056501	0.043244369	0.520651
**hsa-miR-30b**	-0.00264	-0.016210235	0.521528
**hsa-miR-204**	0.002238	-0.011749116	0.531117
**hsa-miR-335**	0.069115	0.04476811	0.532751
**hsa-miR-128a**	-0.07758	-0.063934588	0.535766
**hsa-miR-185**	-0.01811	0.00287811	0.539324
**hsa-miR-27a**	-0.03057	-0.019612343	0.551875
**hsa-miR-218**	0.050686	0.040570403	0.562871
**hsa-miR-30d**	-0.01837	-0.006534557	0.571418
**hsa-miR-26b**	0.000796	-0.009498619	0.573392
**hsa-miR-135b**	0.042795	0.031673726	0.592143
**hsa-miR-1**	-0.04619	-0.035877669	0.609912
**hsa-miR-34a**	0.051988	0.039170629	0.622447
**hsa-miR-140**	-0.01407	-0.027815643	0.625289
**hsa-miR-223**	0.01048	-0.007268749	0.632772
**hsa-miR-7**	0.024443	0.011410001	0.637885
**hsa-miR-181b**	-0.05259	-0.043961334	0.656474
**hsa-miR-302a**	-0.06326	-0.053887197	0.659099
**hsa-miR-152**	-0.00863	0.001116678	0.668968
**hsa-miR-30c**	-0.03353	-0.023140838	0.681944
**hsa-miR-330**	0.037545	0.024842637	0.68261
**hsa-miR-181a**	-0.01327	-0.022272268	0.703619
**hsa-miR-22**	-0.00258	-0.012596816	0.711016
**hsa-miR-122a**	0.017283	0.004962779	0.713419
**hsa-miR-203**	0.036182	0.045141868	0.723871
**hsa-miR-216**	0.018282	0.007990724	0.72893
**hsa-miR-26a**	-0.01051	-0.01912139	0.731239
**hsa-miR-331**	0.033971	0.021436988	0.737696
**hsa-miR-132**	-0.00737	0.002164561	0.738321
**hsa-miR-219**	-0.04135	-0.030528822	0.754777
**hsa-miR-302c**	0.037306	0.029177538	0.75779

**hsa-miR-369**	-0.03314	-0.025902156	0.761978
**hsa-miR-25**	-0.02925	-0.022107675	0.779794
**hsa-let-7e**	-0.03043	-0.025381026	0.781161
**hsa-miR-192**	0.03767	0.050878279	0.788388
**hsa-miR-221**	-0.03948	-0.046922614	0.791044
**hsa-miR-196b**	0.028787	0.019550398	0.795007
**hsa-miR-18**	0.001037	0.009778815	0.804155
**hsa-miR-211**	-0.04794	-0.053747878	0.827562
**hsa-miR-19a**	0.011411	0.007796249	0.838228
**hsa-miR-130b**	-0.01068	-0.01445551	0.843632
**hsa-miR-153**	-0.04832	-0.043148388	0.850756
**hsa-miR-326**	0.006318	0.011391387	0.854501
**hsa-miR-130a**	0.008741	0.006030217	0.885873
**hsa-miR-27b**	-0.02777	-0.025501114	0.898146
**hsa-miR-222**	-0.04129	-0.044783045	0.912954
**hsa-miR-23b**	-0.02475	-0.026858198	0.927022
**hsa-miR-148b**	-0.02997	-0.028115776	0.934489
**hsa-miR-149**	0.035361	0.036663202	0.958672
**hsa-miR-136**	-0.04037	-0.038153745	0.961775
**hsa-miR-338**	-0.00141	-0.002570564	0.967154
**hsa-miR-206**	-0.03592	-0.035230562	0.973471
**hsa-miR-30a-3p**	-0.03125	-0.03072334	0.989228
**hsa-miR-23a**	-0.03212	-0.03185989	0.990595
**hsa-miR-31**	0.034634	0.034918793	0.992607
